# Gene-Specific DNA Methylation Association with Serum Levels of C-Reactive Protein in African Americans

**DOI:** 10.1371/journal.pone.0073480

**Published:** 2013-08-19

**Authors:** Yan V. Sun, Alicia Lazarus, Jennifer A. Smith, Yu-Hsuan Chuang, Wei Zhao, Stephen T. Turner, Sharon L. R. Kardia

**Affiliations:** 1 Department of Epidemiology, Rollins School of Public Health, Emory University, Atlanta, Georgia, United States of America; 2 Department of Biomedical Informatics, School of Medicine, Emory University, Atlanta, Georgia, United States of America; 3 Department of Epidemiology, School of Public Health, University of Michigan, Ann Arbor, Michigan, United States of America; 4 Division of Nephrology and Hypertension, Mayo Clinic, Rochester, Minnesota, United States of America; University of Alabama at Birmingham, United States of America

## Abstract

A more thorough understanding of the differences in DNA methylation (DNAm) profiles in populations may hold promise for identifying molecular mechanisms through which genetic and environmental factors jointly contribute to human diseases. Inflammation is a key molecular mechanism underlying several chronic diseases including cardiovascular disease, and it affects DNAm profile on both global and locus-specific levels. To understand the impact of inflammation on the DNAm of the human genome, we investigated DNAm profiles of peripheral blood leukocytes from 966 African American participants in the Genetic Epidemiology Network of Arteriopathy (GENOA) study. By testing the association of DNAm sites on CpG islands of over 14,000 genes with C-reactive protein (CRP), an inflammatory biomarker of cardiovascular disease, we identified 257 DNAm sites in 240 genes significantly associated with serum levels of CRP adjusted for age, sex, body mass index and smoking status, and corrected for multiple testing. Of the significantly associated DNAm sites, 80.5% were hypomethylated with higher CRP levels. The most significant Gene Ontology terms enriched in the genes associated with the CRP levels were immune system process, immune response, defense response, response to stimulus, and response to stress, which are all linked to the functions of leukocytes. While the CRP-associated DNAm may be cell-type specific, understanding the DNAm association with CRP in peripheral blood leukocytes of multi-ethnic populations can assist in unveiling the molecular mechanism of how the process of inflammation affects the risks of developing common disease through epigenetic modifications.

## Introduction

Inflammation plays a key role in the development of atherosclerosis and cardiovascular diseases [1]. The molecular mechanism underlying atherosclerosis involves the interplay between the immune system and environmental risk factors such as diet, exercise, and smoking. Biomarkers of the immune system, including serum levels of several inflammatory markers, such as C-reactive protein (CRP), fibrinogen, and interleukin 6 are independently associated with cardiovascular diseases (CVD) after adjustment for other known risk factors [2].

Epigenetic modification, through DNA methylation (DNAm) and other molecular mechanisms, can regulate gene expression levels and is an important molecular mechanism underlying disease development. Reports have suggested that epigenetic alteration may lead to the development of immune disorders [3–5]. Further, epigenetic modifications in the immune cells can affect their functionality in inflammatory responses of the human body, which play a key role in developing chronic conditions such as CVD.

CRP is a biomarker of systemic inflammation and a risk factor for the development of inflammation-mediated diseases such as CVD, metabolic syndrome, type 2 diabetes and hypertension [6,7]. The production of CRP in the liver is triggered by cytokines (e.g. IL6 which is secreted by macrophages and T-cells) in response to inflammatory conditions. As a downstream biomarker of inflammatory conditions, CRP also integrates upstream signals from cytokine activation and environmental stimuli. Serum CRP has a long half-life with low circadian variation, and can be easily measured using standardized methods [8,9]. CRP level is heritable [10] and is associated with common genetic variants [6,11,12]. CRP-associated genetic loci have been identified through the genome-wide association study (GWAS) in large sample size in multiple ethnic groups [13,14]. CRP level is also associated with age, sex and environmental factors such as secondhand smoke exposure, air pollution and diet [15]. Epigenetic profile is associated with cigarette smoking [16–18], pesticides [19] and other environmental toxicants [20]. These non-genetic factors can modify the epigenetic profile of genes to alter gene expression levels, potentially leading to changes in disease phenotypes. We hypothesized that the serum CRP level is associated with the epigenetic profile, specifically DNAm, which may represent the joint effect of both genetic and environmental factors.

In this study, we investigated the epigenetic associations of CRP, an inflammatory biomarker of CVD, by measuring over 27,000 DNA methylation (DNAm) sites in peripheral blood leukocytes (PBLs) of 966 African Americans. We identified DNAm sites significantly associated with serum levels of CRP across the genome.

## Materials and Methods

### Sample

The final sample for analysis consisted of 966 African Americans from Jackson Mississippi in the Genetic Epidemiology Network of Arteriopathy (GENOA) study, a community-based study of hypertensive sibships that aims to identify genes influencing blood pressure [21,22]. GENOA data was collected in two phases. Phase I (1996-1999) and Phase II (2000-2004) data consist of demographic information, medical history, clinical characteristics, lifestyle factors, and blood samples for genotyping and biomarker assays. The blood samples and phenotypic data used in this study were collected during GENOA Phase II study. The GENOA study was approved by the Institutional Review Boards of the University of Michigan, Mayo Clinic and Emory University. Each participant gave written informed consent.

### Phenotype Measurement

Age, sex and other phenotypic data were collected from the physical examination and laboratory assessment at the time of the Phase II study visit. Serum CRP levels were measured by a highly sensitive immunoturbidimetric assay on fasting serum samples that had been stored at -80°C and thawed at room temperature [23].

### Genome-wide Methylation Assay

The genomic DNA was extracted from stored PBL samples of 1,008 GENOA Phase II African American (AA) participants from 498 sibships, bisulfite converted and then genotyped for methylation profiling of 27,578 CpG loci using the Illumina Infinium HumanMethylation27 BeadChip (Illumina, San Diego, CA) as previously described [24]. The intensity data of the methylated and unmethylated bead sites from Illumina iScan system were then loaded into the GenomeStudio Methylation Module for analysis. Forty nine samples were excluded from the following analysis due to poor quality of the intensity measurements of control probes. The cleaned data set included DNAm profiles of 972 AA individuals from 493 sibships. After merging with the phenotypic data, 966 AAs had complete phenotypes and DNAm measurements.

There are 56 control probes on each chip representing a) *sample independent* measures of staining, hybridization, target removal, and DNA extension and b) *sample dependent* measures of bisulfite conversion, G/T mismatch, non-polymorphic and negative controls. The sample independent controls allow for the evaluation of the quality of the chip processing steps, while the sample dependent controls allow for the evaluation of the performance across samples. We removed sites with control probe values greater than 4 standard deviations from their mean values. In addition, we developed a normalization scheme to reduce batch and chip effects by linearly regressing the methylated and non-methylated intensity signals onto the set of control probes that are orthogonal (i.e. independent) predictors of the control spot distributions. We also excluded sites that are located on the X and Y chromosomes. Sites that are deemed multimodal based on the Dip Test proposed by Hartigan [25] using a cut-off of p<0.001 on either the methylated or non-methylated signal intensities were set aside for more specialized methods of normalization that take into account their modality. Finally, we flagged the 2,984 sites identified by Chen et al. [26] as having non-specific binding probes (over 10% of the probes map to highly homologous genomic sequences at 40 or more of their base pairs), as well as the 875 sites that have probes overlapping with SNPs reported in dbSNP, which may influence the methylation levels reported by the microarray. We separated out these nonspecific and polymorphic sites after all analyses to aid in interpretation of results. Total number of 22,927 DNAm sites are tested for their associations with serum level of CRP in this study.

We implemented an internal replication design [27,28] to validate our findings of CRP-associated DNA methylation. We took advantage of the sibship-based design of GENOA study and created two datasets, each with 393 unrelated individuals, to test for replication of epigenetic associations with CRP in study groups with similar genetic and environmental backgrounds. We randomly sampled one sibling from each sibship with at least two sibs (n=294) without replacement to create the first dataset (referred to here as Dataset 1). From the remaining participants, we randomly sampled a second sibling from each sibship with at least two sibs to establish the second dataset (referred to here as Dataset 2). The same number of singletons (total n=198) was randomly assigned to each dataset. Therefore, the subjects within each dataset (n=393) were unrelated to each other. Within each of Dataset 1 and Dataset 2, we conducted the multiple linear regression analysis of lnCRP adjusted for the same set of covariates as in the analysis of the pooled 966 samples.

**Table 1:** Summary of demographic variables.

	Females (N=685, 70.9%)	Males (N=281, 29.1%)
	Mean±SD	Mean±SD
Age (yr.)	66.10±7.56	66.70±7.64
BMI^*^ (kg/m^2^)	32.06±6.58	28.98±4.81
SBP (mm Hg)	140.88±21.62	138.05±20.75
DBP^*^ (mm Hg)	77.52±10.83	80.39±11.06
CRP^*^ (mg/L), median (Q1, Q3)	0.38 (0.19, 0.80)	0.27 (0.12, 0.58)

* statistically different between males and females (p-value < 0.05).

**Table 2:** Summary of the 30 most significant CRP-associated DNA methylation sites.

DNAm	Gene	Chr.	Location^*^ (bp)	Strand	Beta (SE)	P-value	P-value2^**^	ds1 P-value	ds2 P-value
cg07073964	KLK10	19	649371	-	-4.12 (0.58)	5.85×10^-12^	4.43×10^-12^	3.51×10^-3^	1.81×10^-6^
cg09358725	LMO2	11	33870664	-	-3.60 (0.52)	1.69×10^-11^	2.21×10^-11^	1.83×10^-4^	1.80×10^-5^
cg04121771	TM4SF4	3	150674314	+	-4.42 (0.68)	2.05×10^-10^	6.88×10^-11^	2.25×10^-4^	1.46×10^-4^
cg08458487	SFTPD	10	81699171	-	-2.79 (0.43)	2.26×10^-10^	3.07×10^-10^	4.09×10^-4^	3.48×10^-5^
cg09305224	FUT7	9	139047066	-	-3.38 (0.52)	2.48×10^-10^	2.76×10^-10^	6.95×10^-4^	3.82×10^-5^
cg00645579	IRF7	11	607140	-	-3.80 (0.59)	2.94×10^-10^	2.85×10^-10^	8.55×10^-4^	7.06×10^-6^
cg05556717	CCL26	7	75257240	-	-3.32 (0.52)	3.94×10^-10^	3.12×10^-10^	2.13×10^-4^	8.39×10^-7^
cg17496921	TSPAN16	19	11267993	+	-2.94 (0.46)	4.97×10^-10^	4.78×10^-10^	5.68×10^-4^	1.12×10^-4^
cg03801286	KCNE1	21	34806378	-	-2.62 (0.41)	5.61×10^-10^	5.74×10^-10^	2.52×10^-4^	2.34×10^-4^
cg21969640	GPR84	12	53043844	-	-3.08 (0.49)	6.03×10^-10^	6.28×10^-10^	2.69×10^-4^	4.88×10^-5^
cg05501357	HIPK3	11	33264845	+	-3.35 (0.53)	6.29×10^-10^	7.41×10^-10^	8.58×10^-4^	1.83×10^-4^
cg03600318	SFTPD	10	81698971	-	-3.58 (0.57)	7.01×10^-10^	6.80×10^-10^	1.65×10^-4^	1.99×10^-3^
cg18084554	ARID3A	19	880046	+	-2.68 (0.43)	7.91×10^-10^	1.03×10^-9^	1.19×10^-4^	7.68×10^-5^
cg06625767	F12	5	176769301	-	-2.80 (0.45)	1.04×10^-9^	1.07×10^-9^	3.82×10^-3^	3.38×10^-5^
cg15248035	CCIN	9	36159949	+	-2.62 (0.42)	1.22×10^-9^	1.36×10^-9^	7.39×10^-4^	1.79×10^-4^
cg05546038	NOL3	16	65764534	+	-3.96 (0.64)	1.40×10^-9^	1.58×10^-9^	2.21×10^-5^	2.23×10^-3^
cg09303642	NFE2	12	52977085	-	-2.70 (0.44)	1.60×10^-9^	2.28×10^-9^	1.94×10^-4^	1.01×10^-4^
cg03330678	SEPT9	17	72827828	+	-2.62 (0.43)	1.69×10^-9^	2.27×10^-9^	1.62×10^-3^	3.57×10^-5^
cg17753124	IER2	19	13120872	+	-3.34 (0.54)	1.72×10^-9^	1.31×10^-9^	1.59×10^-3^	3.50×10^-4^
cg22242539	SERPINF1	17	1611970	+	-3.25 (0.53)	2.08×10^-9^	1.86×10^-9^	2.31×10^-4^	8.51×10^-4^
cg17166812	NDUFS2	1	159436198	+	-3.89 (0.64)	2.28×10^-9^	2.30×10^-9^	4.44×10^-3^	1.82×10^-4^
cg22266967	S100P	4	6746599	+	-2.84 (0.47)	2.29×10^-9^	2.39×10^-9^	2.58×10^-3^	6.12×10^-5^
cg12380764	IL19	1	205037818	+	-2.93 (0.48)	2.35×10^-9^	2.93×10^-9^	2.38×10^-4^	4.40×10^-4^
cg10275770	ICAM2	17	59437937	-	-3.64 (0.60)	2.51×10^-9^	2.21×10^-9^	5.35×10^-4^	1.06×10^-3^
cg21492378	CEP1	9	122890100	+	-3.68 (0.60)	2.53×10^-9^	2.08×10^-9^	1.29×10^-4^	9.80×10^-6^
cg22381196	DHODH	16	70598877	+	-2.24 (0.37)	2.99×10^-9^	3.53×10^-9^	3.67×10^-4^	7.47×10^-4^
cg23140706	NFE2	12	52975545	-	-4.22 (0.70)	2.99×10^-9^	3.90×10^-9^	6.65×10^-4^	1.32×10^-3^
cg20283107	FAM91A1	8	124858150	+	-3.65 (0.60)	3.12×10^-9^	3.57×10^-9^	2.48×10^-4^	4.31×10^-6^
cg27606341	FYB	5	39255389	-	-2.55 (0.42)	3.21×10^-9^	3.31×10^-9^	1.67×10^-4^	8.62×10^-4^
cg26861460	PARVG	22	42906788	+	-2.90 (0.48)	3.27×10^-9^	3.42×10^-9^	3.68×10^-4^	1.29×10^-4^

* Chromosomal location is based on NCBI build 36.1.

** P-value2 was from the sensitivity analysis adjusted for age, gender, BMI, current smoking and hypertension status.

An internal replication was conducted by randomly splitting 966 samples into two mutually exclusive subsets (see details in the Methods), ds1 and ds2, each with 393 unrelated individuals. The beta coefficients and p-values were calculated using linear regression models for each subset.

### Statistical Methods

Serum CRP level was transformed with the natural logarithm (ln) because of its skewed distribution. We forced age and sex in the multivariable regression models and identified body mass index (BMI) as significantly associated with lnCRP. LnCRP values were adjusted for age, sex, BMI and cigarette smoking (current smoker) in a multivariable regression model to identify CRP-associated DNAm sites in the association analyses. The “current smoker” was defined as a binary variable indicating if an individual had smoked within the past year or not. To identify sex-specific epigenetic associations with serum CRP, we evaluated the sex-DNAm interaction term in the multivariable-adjusted model for each DNAm site significantly associated with CRP level.

Based on chromosomal location (NCBI 36.1), we identified CRP-associated genes on human autosomes. Bonferroni corrected p-value of 0.05 (nominal p-value of 2.18×10^-6^) was applied to adjust for multiple testing of 22,927 autosomal DNAm sites. We used gProfile [29] to estimate the enrichment of Gene Ontology (GO) terms and identify the over-represented GO terms linked to the CRP-associated genes.

Linear mixed models were implemented in a multiple regression framework to adjust for the relatedness of the studied individuals. The sibship relationship was modeled as a random effect using the sibship identifiers. All statistical analyses were performed with R statistical environment version 2.11.1 from R Project (http://www.r-project.org/). The authors had full access to the data and take responsibility for its integrity.

## Results

Table 1 summarizes the key phenotypes of the GENOA AA participants. The final analysis dataset included 966 AA participants from 492 sibships. This AA sample had various sizes of sibships with up to 10 siblings per sibship, with average sibship size of 1.96 siblings. About one fifth of the participants were singletons without any relatives included in the sample. Within the sample, 80.1% of males and 83.5% of females were diagnosed as having hypertension at the time of visit. Females had significantly higher BMI than males in this study. Serum CRP levels for female participants (median of 0.38 mg/L) were significantly higher than that of male participants (median of 0.27 mg/L).

**Figure 1:**
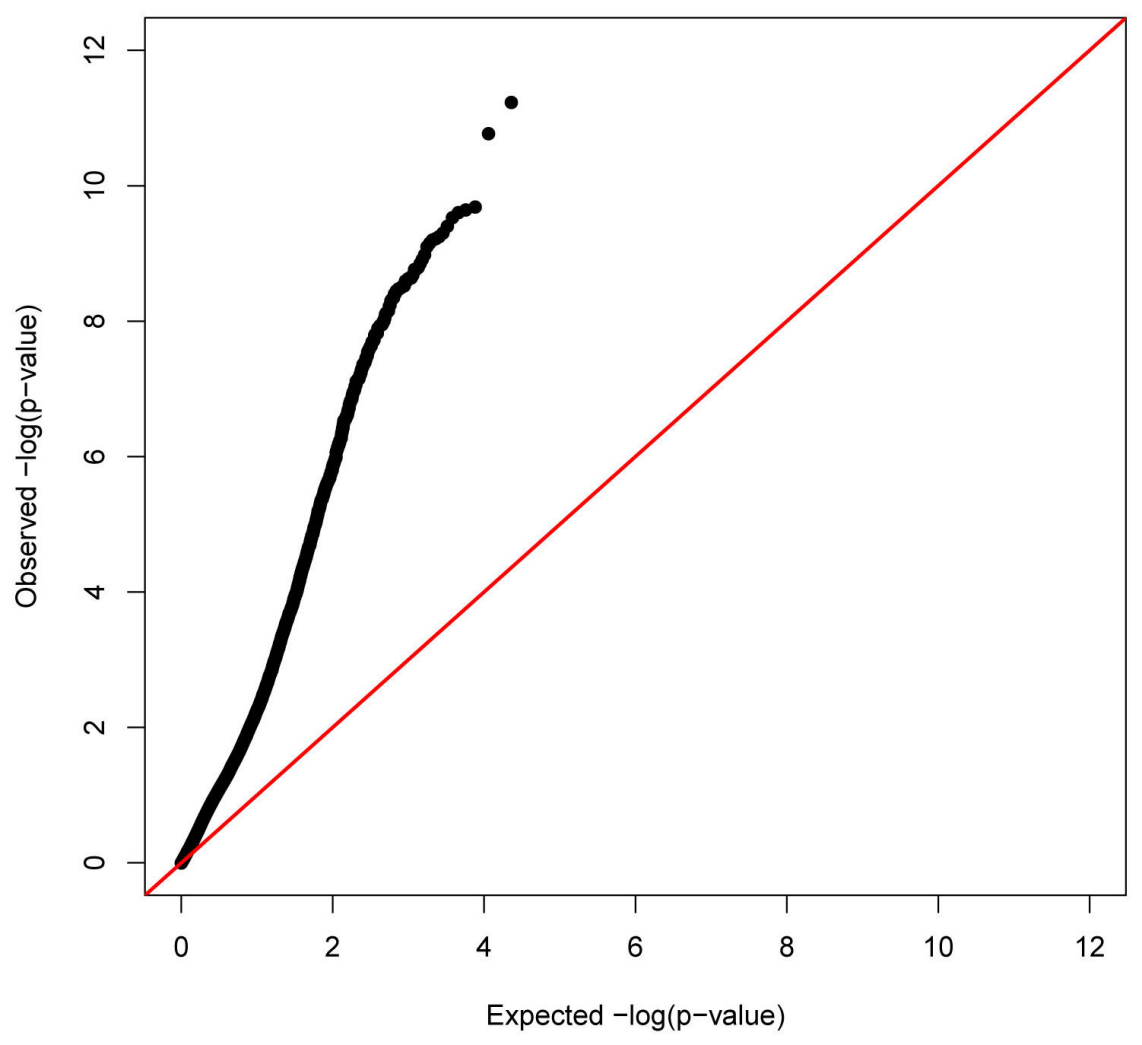
Quantile-quantile plot of DNAm association with logCRP.

**Figure 2:**
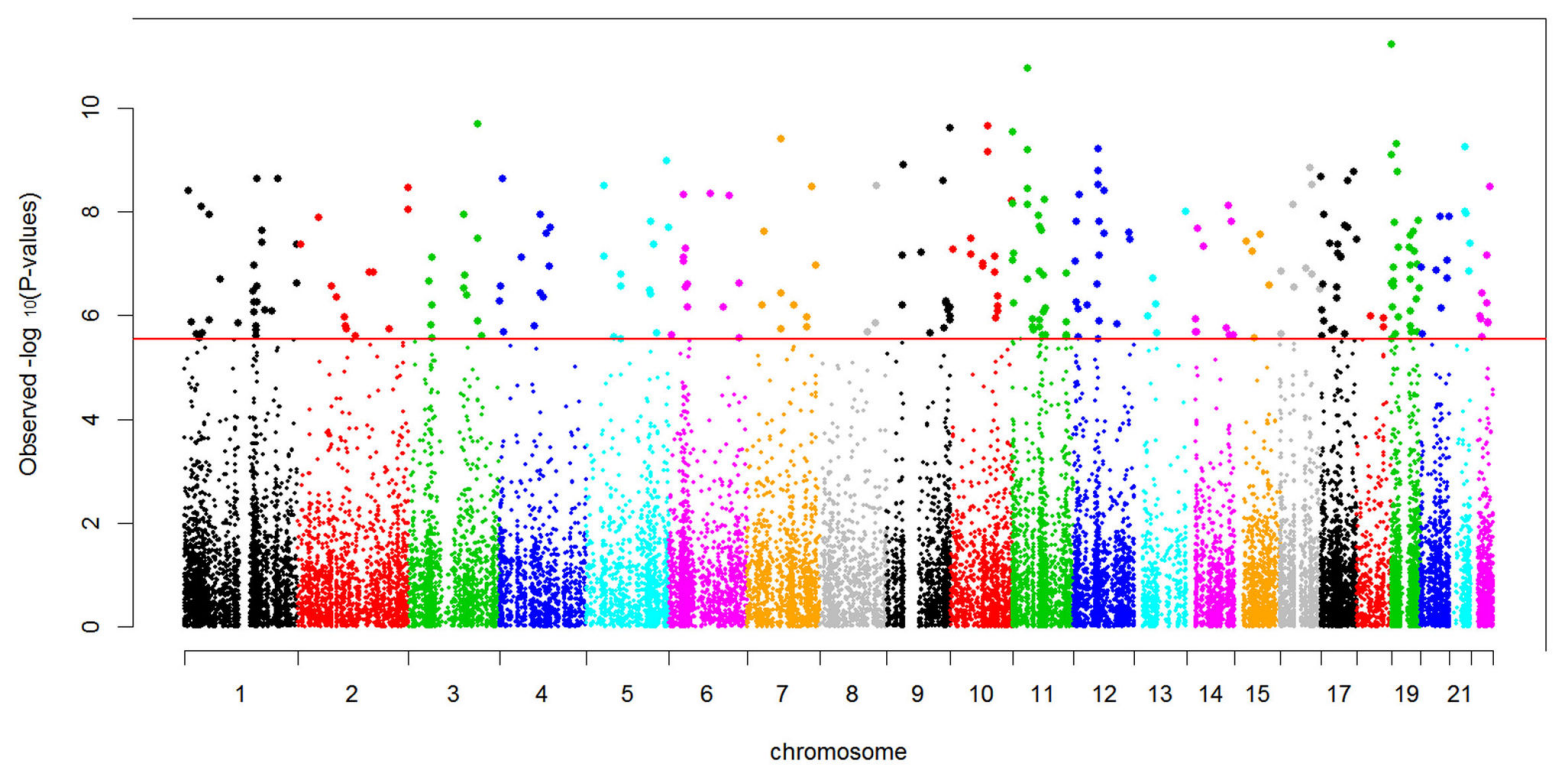
Manhattan plot of methylome-wide association with log CRP in 966 African Americans, adjusted for age, gender, BMI and cigarette smoking.

Using stringent Bonferroni correction for multiple testing in the analyses of linear mixed models to identify CRP-associated DNAm sites influencing natural log-transformed CRP (lnCRP), we identified 257 autosomal DNAm sites significantly associated with serum CRP levels (Table S1). The distribution of p-values was substantially inflated as shown in Figure 1 (Quantile-quantile plot). These DNAm sites mapped to 240 human genes across all 22 autosomes (Figure 2). Among 257 CRP-associated DNAm sites, 80.5% (207 out of 257 sites) exhibited an inverse correlation of greater methylation with lower level of CRP. Among the thirty most significant DNAm associations with CRP (Table 2), all DNAm sites exhibited a negative correlation of greater methylation with lower level of CRP. Using the two subsets each with 393 unrelated individuals, we confirmed that the top 30 associations (Table 2) have significant associations at the nominal p-value threshold correcting for 30 tests (1.67×10^-3^) in at least one subset (24 out of 30 have the significant p-values in both subset). By testing the sex-DNAm interaction term in a multiple regression model for each DNAm site, the most significant test had a p-value of 1.34×10^-4^. After correction of multiple testing, none of the interactions between sex and DNAm had statistically significant effects on serum CRP level.

There were 240 unique genes annotated to the 257 CRP-associated DNAm sites. By searching the Gene Ontology (GO) database of *homo sapiens* through g: Profiler [29], we found five GO terms most over-represented among these genes with p-value less than 5×10^-6^ (Table 3). All five over-represented GO terms belonged to the Biological Process of “immune system process” or “response to stimulus”. There were 61 genes annotated to the GO category of “immune system process” and 126 genes annotated to “response to stimulus” (listed in Table S2).

**Table 3:** Over-represented GO terms for genes with CRP-associated DNAm sites.

GO Term ID	GO term name	GO Domain^*^	Number of Genes	p-value
GO:0002376	immune system process	BP	61	1.09×10^-12^
GO:0050896	response to stimulus	BP	126	1.84×10^-9^
GO:0006950	response to stress	BP	68	6.28×10^-7^
GO:0006952	defense response	BP	39	2.74×10^-6^
GO:0006955	immune response	BP	46	1.16×10^-10^

* BP: Biological Process

**Figure 3:**
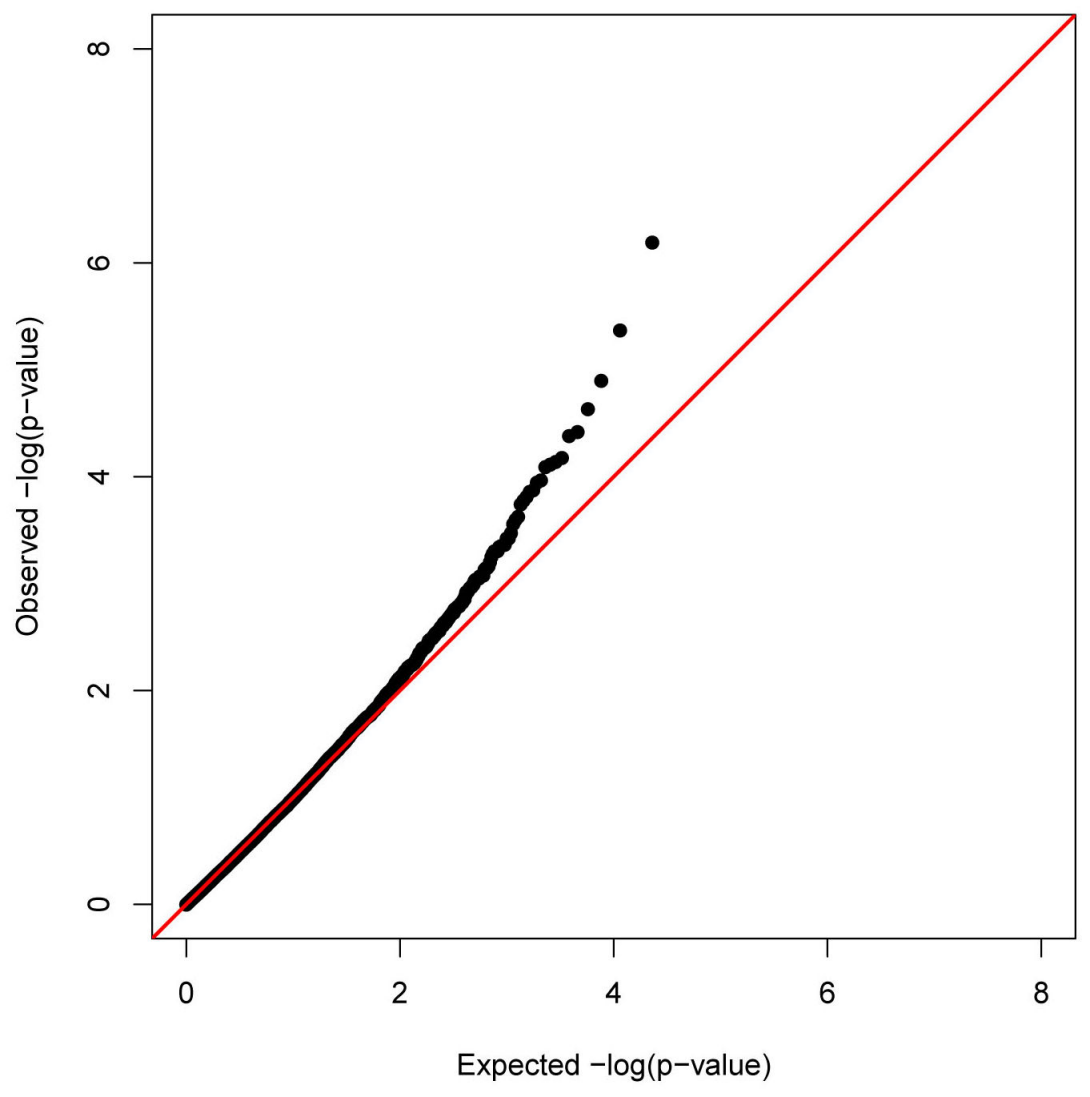
Quantile-quantile plot of DNAm association with lnCRP adjusted for top three principal components of the DNAm data.

We applied a principal component analysis (PCA) to adjust for the inflation due to unmeasured confounders [30]. Using the top three principal components of the DNAm data, the inflation of the low p-values was well controlled with an inflation factor of 0.95 (Figure 3). Two DNAm sites, cg05316065 (*MLZE*) and cg27637521 (*SOCS3*), remained genome-wide significant with FDR q-values of 0.015 (nominal p-value of 6.44×10^-7^) and 0.049 (nominal p-value of 4.27×10^-6^) respectively. The most significant CRP-associated DNAm site for the analysis without PCA adjustment, cg07073964 (*KLK10*), was the fourth most significant DNAm site, with FDR q-value of 0.133 (nominal p-value of 2.33×10^-5^).

## Discussion

Using a large sample of AAs, we identified widespread CRP-associated DNAm sites across all 22 autosomes. These DNAm sites are mapped to a set of genes highly enriched for cellular defense mechanisms, immune responses and stress responses (Table 3), which are the key functions of leukocytes in the blood and are related to inflammatory responses.

The most significant CRP-associated DNAm (cg07073964) is located close to kallikrein-related peptidase 10 (*KLK10*) gene on chromosome 19. *KLK10* encodes for a secreted protein, and is expression in many human tissues [31]. *KLK10* may function as a tumor suppressor gene. The expression of *KLK10* is down-regulated in several cancers (e.g. breast, prostate and non-small cell lung cancer, and hepatocellular carcinoma), and up-regulated in colorectal cancer, ovarian cancer, pancreatic ductal adenocarcinoma and uterine papillary serous carcinoma. The level of DNA methylation of *KLK10* gene is reversely associated with the gene expression levels in human tissues [32,33]. In a recent study, expression of Klk10 was down-regulated in mouse carotid artery after a partial ligation which rapidly induces atherosclerosis [34]. In the same study, Lmo4 gene showed higher expression in mouse aortic arch after ligation, and in human coronary endothelium exposed to oscillatory shear [34]. The second most significant CRP-associated DNAm (cg09358725) is located on the promoter of LIM domain only 2 (*LMO2*) gene on chromosome 11. Another DNAm site (cg11822932) of *LMO2* gene was also strongly associated with CRP levels (ranking 44^th^, Bonferroni corrected p-value of 1.65×10^-4^). The *LMO2* protein is a highly conserved transcription factor and has a crucial role in early stage of hematopoietic development [35]. All cellular blood components including leukocytes are derived from hematopoietic stem cells. The modification of *LMO2* expression level may have broad impact on the cellular functions of the blood cells.

There are several gene families related to the immune system that are enriched in the gene set of CRP-associated DNAm. Six immunorecepter (CD) genes, *CD1D*, *CD7*, *CD22*, *CD27*, *CD59* and *CD82*, and five interleukin and receptor genes, *IL1R2*, *IL2RΑ*, *IL19*, *IL21R*, *IL32* were identified by the epigenetic association analysis. The methylation sites in five G-protein coupled receptor (GPR) genes, *GPR21*, *GPR65*, *GPR81*, *GPR84* and *GPR171*, were also found to be associated with CRP. The GPR gene family encodes receptors spanning the cellular membrane for signal transduction. GPRs play important roles in vision, smell, immune systems, and the autonomic nervous system, and are major drug targets of numerous human diseases [36].

To further understand the potential functional impact, we calculated the range of methylation difference of the most significant CRP-related CpG site, cg07073964 (*KLK10*). In the lowest quartile of serum level of CRP, the mean beta-value is 0.52, 6% higher than that in the highest quartile of CRP levels (mean beta-value of 0.49). Without the direct measurement of gene expression levels, we could not assess the impact of the methylation difference of cg07073964 on the transcription of *KLK10*. Due to the limited amount of change in DNA methylation, the functional consequence of the individual CRP-associated DNAm might not be substantial. However, the combination of a number of DNAm sites from multiple related pathways may have a much larger impact on the molecular function.

Despite the discovery that DNAm measured by LINE-1 repetitive elements, a proxy for global DNAm measurements, in PBLs of 593 elderly white males were not found to be significantly associated with CRP levels [37], DNAm has been associated with CRP levels at specific sites. Uddin et al. found a significant inverse correlation between methylation of the *IL6* gene, involved in the inflammatory response, and serum levels of CRP in 33 individuals with a lifetime history of depression and 67 non-depressed controls [38]. Two DNAm sites located close to the *IL6* gene, cg15703690 and cg01770232, were measured in our sample of AAs. Neither of these sites showed significant association with serum levels of CRP after correction of multiple testing. However, cg15703690 was inversely correlated with CRP levels with a nominal p-value of 0.0017 after adjustment for age, sex, BMI and smoking status.

Nearly 80% of the significant gene-specific DNAm sites exhibited an inverse correlation between hypomethylation and higher levels of CRP in our sample. Interestingly, we observed a similar trend between gene-specific DNAm and age in this AA sample: most age-related DNAm sites are hypomethylated in older age (unpublished). Age-related hypomethylation has also been previously reported in other ethnic groups [39]. A similar pattern of modifications of DNAm on CpG islands between chronic aging and inflammatory markers may indicate shared molecular mechanisms underlying chronic diseases through epigenetic changes.

The GENOA participants were enriched for hypertensives. Thus, this study’sAA sample is a high-risk cohort for cardiovascular diseases. The epigenetic associations with CRP identified in this study may not be generalizable to the non-diseased population. However, our epigenome-wide association study (EWAS) may greatly contribute toward the understanding of the disease etiology among AAs, who have the highest prevalence of hypertension among racial groups in the U.S. and are under-represented in genetic and epigenetic research. To address the potential impact of hypertension, we adjusted for hypertension status, in addition to age, gender, BMI and cigarette smoking in a sensitivity analysis. The test statistics of the DNAm sites between the primary model and the model adjusted for hypertension were consistent (R^2^=0.998), and the p-values of the top 30 sites were similarly significant between the two models (table 2). These results suggest that hypertension status did not affect the findings of the CRP-associated DNAm in the primary analysis.

Significant findings in epigenetic association studies should be validated in replicate samples as recommended for genetic association studies [40] in order to prevent false-positive results which can be caused by the variation in the biosample/experimental procedure [41] or analytical bias [42]. In this study of epigenetic associations with serum levels of CRP, we were not able to replicate the significant associations with CRP because we could not locate an independent sample with measurements of both CRP and methylome. Instead, we capitalized on the sibship structure of our data to create two replicate sets of unrelated individuals (one sib in unrelated subset 1, second sib in unrelated subset 2) to perform replication analysis of significant epigenetic associations. Among the top 30 associations, the test statistics were consistent between the two subsets and were mostly significant. Although this internal replication partially addressed some issues that may cause false-positive findings, future studies on independent populations are needed to fully validate the findings of epigenetic associations with CRP. We also observed substantial inflation of low p-values in the primary EWAS analysis. Such inflation in EWAS can be caused by unmeasured confounders (e.g. batch effect) or wide-spread associations on the methylome (e.g. age-related DNAm). Because of the overall inflation, we might not have fully controlled the type-1 error. By adjusting for the top three PCs of the DNAm data and multiple testing in a secondary analysis, we were able to fully control the inflation and identified two significant CRP-associated DNAm sites. However, without knowing the true underlying distribution of the epigenome-wide association, we could have over-corrected for the inflation and potentially increased the type-II error.

DNAm profiles are tissue and cell type-specific [24,43]. DNA methylation profiles have been commonly studied in PBLs due to the easy access to the biosample. The choice of PBLs is meaningful to study certain environmental exposures such as smoking, and chronic conditions involving the circulation and immune system. However, since PBLs comprise a mixture of multiple cell types, it is possible that the results reported here and elsewhere reflect inflammation-related DNAm changes that influence a single cell type component of PBLs. Since different DNAm profiles have been observed in distinct leukocyte subtypes using dozens of samples [44,45], the association between DNA methylation and CRP can be confounded by differences in the proportion of leukocyte subtypes between samples. Therefore we cannot rule out the impact of shift of cell populations on DNAm. Future studies of epigenetic associations in a single targeted cell population would be valuable for identifying cell-type specific associations between DNAm and CRP and other inflammatory biomarkers.

In conclusion, we identified over two hundred genes containing CRP-associated DNAm sites. The results highlight immune response and other cellular response genes involved in the regulation of chronic inflammation. Furthermore, the epigenetic variants associated with CRP levels do not directly overlap with the genetic variants influencing CRP levels, but they are involved in common pathways and gene families related to inflammation. Although we observed strong gene-specific epigenetic associations with CRP levels, for each identified gene, the underlying molecular mechanisms related to inflammation are largely unknown. These epigenetic modifications can be the triggers or consequences of inflammatory responses. Future replication studies are warranted to confirm the association between the DNA methylation sites and serum level of CRP. 

## Supporting Information

Table S1 **Summary of DNA methylation sites significantly associated with serum levels of CRP.**
(PDF)

Table S2 **List of CRP-associated genes annotated to two GO terms “immune system process” and “response to stimulus.**
(XLS)
