# Supplementary material for: Gene-Specific DNA Methylation Association with Serum Levels of C-Reactive Protein in African Americans
Source: PLoS One. 2013 Aug 19;8(8):e73480. doi: 10.1371/journal.pone.0073480 (PMC3747126; doi:10.1371/journal.pone.0073480)
Supplement: Table S1 — Summary of DNA methylation sites significantly associated with serum levels of CRP. (PDF) [file pone.0073480.s001.pdf]

Supplement Table 1. The summary of 257 significant DNA methylation sites associated with CRP levels

| no. | DNAm | Gene | Chr. | Location* (bp) | Strand | Beta (SE) | P-value | Bonferroni P-value |
|---|---|---|---|---|---|---|---|---|
| 1 | cg07073964 | KLK10 | 19 | 649371 | - | -4.12 (0.58) | $5.85 \times 10^{-12}$ | $1.34 \times 10^{-7}$ |
| 2 | cg09358725 | LMO2 | 11 | 33870664 | - | -3.60 (0.52) | $1.69 \times 10^{-11}$ | $3.88 \times 10^{-7}$ |
| 3 | cg04121771 | TM4SF4 | 3 | 150674314 | + | -4.42 (0.68) | $2.05 \times 10^{-10}$ | $4.70 \times 10^{-6}$ |
| 4 | cg08458487 | SFTPD | 10 | 81699171 | - | -2.79 (0.43) | $2.26 \times 10^{-10}$ | $5.19 \times 10^{-6}$ |
| 5 | cg09305224 | FUT7 | 9 | 139047066 | - | -3.38 (0.52) | $2.48 \times 10^{-10}$ | $5.67 \times 10^{-6}$ |
| 6 | cg00645579 | IRF7 | 11 | 607140 | - | -3.80 (0.59) | $2.94 \times 10^{-10}$ | $6.75 \times 10^{-6}$ |
| 7 | cg05556717 | CCL26 | 7 | 75257240 | - | -3.32 (0.52) | $3.94 \times 10^{-10}$ | $9.04 \times 10^{-6}$ |
| 8 | cg17496921 | TSPAN16 | 19 | 11267993 | + | -2.94 (0.46) | $4.97 \times 10^{-10}$ | $1.14 \times 10^{-5}$ |
| 9 | cg03801286 | KCNE1 | 21 | 34806378 | - | -2.62 (0.41) | $5.61 \times 10^{-10}$ | $1.29 \times 10^{-5}$ |
| 10 | cg21969640 | GPR84 | 12 | 53043844 | - | -3.08 (0.49) | $6.03 \times 10^{-10}$ | $1.38 \times 10^{-5}$ |
| 11 | cg05501357 | HIPK3 | 11 | 33264845 | + | -3.35 (0.53) | $6.29 \times 10^{-10}$ | $1.44 \times 10^{-5}$ |
| 12 | cg03600318 | SFTPD | 10 | 81698971 | - | -3.58 (0.57) | $7.01 \times 10^{-10}$ | $1.61 \times 10^{-5}$ |
| 13 | cg18084554 | ARID3A | 19 | 880046 | + | -2.68 (0.43) | $7.91 \times 10^{-10}$ | $1.81 \times 10^{-5}$ |
| 14 | cg06625767 | F12 | 5 | 176769301 | - | -2.80 (0.45) | $1.04 \times 10^{-9}$ | $2.39 \times 10^{-5}$ |
| 15 | cg15248035 | CCIN | 9 | 36159949 | + | -2.62 (0.42) | $1.22 \times 10^{-9}$ | $2.79 \times 10^{-5}$ |
| 16 | cg05546038 | NOL3 | 16 | 65764534 | + | -3.96 (0.64) | $1.40 \times 10^{-9}$ | $3.21 \times 10^{-5}$ |
| 17 | cg09303642 | NFE2 | 12 | 52977085 | - | -2.70 (0.44) | $1.60 \times 10^{-9}$ | $3.68 \times 10^{-5}$ |
| 18 | cg03330678 | SEPT9 | 17 | 72827828 | + | -2.62 (0.43) | $1.69 \times 10^{-9}$ | $3.88 \times 10^{-5}$ |
| 19 | cg17753124 | IER2 | 19 | 13120872 | + | -3.34 (0.54) | $1.72 \times 10^{-9}$ | $3.94 \times 10^{-5}$ |
| 20 | cg22242539 | SERPINF1 | 17 | 1611970 | + | -3.25 (0.53) | $2.08 \times 10^{-9}$ | $4.76 \times 10^{-5}$ |
| 21 | cg17166812 | NDUFS2 | 1 | 159436198 | + | -3.89 (0.64) | $2.28 \times 10^{-9}$ | $5.24 \times 10^{-5}$ |
| 22 | cg22266967 | S100P | 4 | 6746599 | + | -2.84 (0.47) | $2.29 \times 10^{-9}$ | $5.25 \times 10^{-5}$ |
| 23 | cg12380764 | IL19 | 1 | 205037818 | + | -2.93 (0.48) | $2.35 \times 10^{-9}$ | $5.38 \times 10^{-5}$ |
| 24 | cg10275770 | ICAM2 | 17 | 59437937 | - | -3.64 (0.60) | $2.51 \times 10^{-9}$ | $5.76 \times 10^{-5}$ |
| 25 | cg21492378 | CEP1 | 9 | 122890100 | + | -3.68 (0.60) | $2.53 \times 10^{-9}$ | $5.79 \times 10^{-5}$ |
| 26 | cg22381196 | DHODH | 16 | 70598877 | + | -2.24 (0.37) | $2.99 \times 10^{-9}$ | $6.85 \times 10^{-5}$ |
| 27 | cg23140706 | NFE2 | 12 | 52975545 | - | -4.22 (0.70) | $2.99 \times 10^{-9}$ | $6.86 \times 10^{-5}$ |
| 28 | cg20283107 | FAM91A1 | 8 | 124858150 | + | -3.65 (0.60) | $3.12 \times 10^{-9}$ | $7.15 \times 10^{-5}$ |
| 29 | cg27606341 | FYB | 5 | 39255389 | - | -2.55 (0.42) | $3.21 \times 10^{-9}$ | $7.36 \times 10^{-5}$ |
| 30 | cg26861460 | PARVG | 22 | 42906788 | + | -2.90 (0.48) | $3.27 \times 10^{-9}$ | $7.49 \times 10^{-5}$ |
| 31 | cg18463686 | CLEC5A | 7 | 141293159 | - | -3.21 (0.53) | $3.32 \times 10^{-9}$ | $7.62 \times 10^{-5}$ |
| 32 | cg16967583 | AGXT | 2 | 241456532 | + | -3.02 (0.50) | $3.47 \times 10^{-9}$ | $7.95 \times 10^{-5}$ |
| 33 | cg25600606 | HIPK3 | 11 | 33264921 | + | -2.58 (0.43) | $3.54 \times 10^{-9}$ | $8.13 \times 10^{-5}$ |
| 34 | cg16097772 | LYZ | 12 | 68028499 | + | -3.02 (0.50) | $3.84 \times 10^{-9}$ | $8.81 \times 10^{-5}$ |
| 35 | cg11283860 | SLC45A1 | 1 | 8195939 | + | -2.47 (0.41) | $3.95 \times 10^{-9}$ | $9.05 \times 10^{-5}$ |
| 36 | cg00333528 | GABRR1 | 6 | 89984204 | - | -3.17 (0.53) | $4.50 \times 10^{-9}$ | $1.03 \times 10^{-4}$ |
| 37 | cg08399444 | GSG1 | 12 | 13139815 | - | -2.95 (0.49) | $4.62 \times 10^{-9}$ | $1.06 \times 10^{-4}$ |

| | | | | | | | | | |
|---|---|---|---|---|---|---|---|---|---|
| 38 | cg24211388 | AIF1 | 6 | 31690816 | + | -2.53 | (0.42) | $4.76 \times 10^{-9}$ | $1.09 \times 10^{-4}$ |
| 39 | cg22825487 | VNN3 | 6 | 133096876 | - | -3.54 | (0.59) | $4.99 \times 10^{-9}$ | $1.14 \times 10^{-4}$ |
| 40 | cg25634666 | FOLR3 | 11 | 71524436 | + | -2.62 | (0.44) | $5.74 \times 10^{-9}$ | $1.32 \times 10^{-4}$ |
| 41 | cg22045288 | C10orf91 | 10 | 134108411 | + | -3.50 | (0.59) | $6.00 \times 10^{-9}$ | $1.38 \times 10^{-4}$ |
| 42 | cg22016649 | PNPLA2 | 11 | 808917 | + | -3.58 | (0.61) | $7.00 \times 10^{-9}$ | $1.60 \times 10^{-4}$ |
| 43 | cg11822932 | LMO2 | 11 | 33870292 | - | -3.02 | (0.51) | $7.19 \times 10^{-9}$ | $1.65 \times 10^{-4}$ |
| 44 | cg02656594 | IL21R | 16 | 27319997 | + | -3.03 | (0.51) | $7.36 \times 10^{-9}$ | $1.69 \times 10^{-4}$ |
| 45 | cg14974772 | FBLN5 | 14 | 91484178 | - | -3.49 | (0.59) | $7.53 \times 10^{-9}$ | $1.73 \times 10^{-4}$ |
| 46 | cg07285167 | CSF3R | 1 | 36721568 | - | -2.73 | (0.46) | $7.88 \times 10^{-9}$ | $1.81 \times 10^{-4}$ |
| 47 | cg00795812 | PDCD1 | 2 | 242450682 | - | 3.36 | (0.57) | $8.96 \times 10^{-9}$ | $2.06 \times 10^{-4}$ |
| 48 | cg17356733 | IFNGR2 | 21 | 33696497 | + | -2.40 | (0.41) | $9.82 \times 10^{-9}$ | $2.25 \times 10^{-4}$ |
| 49 | cg07218880 | UPF3A | 13 | 114064381 | + | 3.01 | (0.52) | $9.94 \times 10^{-9}$ | $2.28 \times 10^{-4}$ |
| 50 | cg00673191 | DOPEY2 | 21 | 36458793 | + | -3.44 | (0.59) | $1.06 \times 10^{-8}$ | $2.44 \times 10^{-4}$ |
| 51 | cg26661623 | ASGR2 | 17 | 6959986 | - | -3.66 | (0.63) | $1.12 \times 10^{-8}$ | $2.56 \times 10^{-4}$ |
| 52 | cg07958192 | TIGD2 | 4 | 90252997 | + | -3.54 | (0.61) | $1.12 \times 10^{-8}$ | $2.56 \times 10^{-4}$ |
| 53 | cg10266490 | ACOT11 | 1 | 54786297 | + | -3.38 | (0.58) | $1.13 \times 10^{-8}$ | $2.60 \times 10^{-4}$ |
| 54 | cg02863947 | NR1I2 | 3 | 120981880 | + | -2.69 | (0.46) | $1.15 \times 10^{-8}$ | $2.64 \times 10^{-4}$ |
| 55 | cg23547429 | SLC43A3 | 11 | 56951601 | - | -3.11 | (0.54) | $1.18 \times 10^{-8}$ | $2.70 \times 10^{-4}$ |
| 56 | cg23889010 | SLPI | 20 | 43316404 | - | -3.04 | (0.52) | $1.22 \times 10^{-8}$ | $2.79 \times 10^{-4}$ |
| 57 | cg09868035 | C20orf135 | 20 | 61962518 | + | -3.52 | (0.61) | $1.25 \times 10^{-8}$ | $2.86 \times 10^{-4}$ |
| 58 | cg27485921 | ATP6V1E2 | 2 | 46600883 | - | -2.83 | (0.49) | $1.27 \times 10^{-8}$ | $2.92 \times 10^{-4}$ |
| 59 | cg14511156 | OSCAR | 19 | 59295936 | - | -3.57 | (0.62) | $1.50 \times 10^{-8}$ | $3.45 \times 10^{-4}$ |
| 60 | cg17813891 | EVL | 14 | 99601789 | + | 3.03 | (0.53) | $1.52 \times 10^{-8}$ | $3.49 \times 10^{-4}$ |
| 61 | cg22630748 | INHBE | 12 | 56135137 | + | -3.81 | (0.66) | $1.53 \times 10^{-8}$ | $3.52 \times 10^{-4}$ |
| 62 | cg11384427 | TNFRSF7 | 12 | 6423846 | + | 3.61 | (0.63) | $1.55 \times 10^{-8}$ | $3.54 \times 10^{-4}$ |
| 63 | cg00145118 | GNPDA1 | 5 | 141374115 | - | -3.63 | (0.63) | $1.55 \times 10^{-8}$ | $3.55 \times 10^{-4}$ |
| 64 | cg09001777 | FUT3 | 19 | 5802504 | - | -3.24 | (0.56) | $1.61 \times 10^{-8}$ | $3.70 \times 10^{-4}$ |
| 65 | cg04988978 | MPO | 17 | 53714577 | - | -3.57 | (0.62) | $1.86 \times 10^{-8}$ | $4.27 \times 10^{-4}$ |
| 66 | cg04353769 | MS4A6A | 11 | 59708133 | - | -2.94 | (0.51) | $1.94 \times 10^{-8}$ | $4.44 \times 10^{-4}$ |
| 67 | cg16361890 | LTC4S | 5 | 179153151 | + | -3.59 | (0.63) | $1.97 \times 10^{-8}$ | $4.51 \times 10^{-4}$ |
| 68 | cg03886110 | PECAM1 | 17 | 59817773 | - | -2.89 | (0.51) | $1.98 \times 10^{-8}$ | $4.53 \times 10^{-4}$ |
| 69 | cg17714799 | CASP6 | 4 | 110844529 | - | -2.97 | (0.52) | $2.01 \times 10^{-8}$ | $4.60 \times 10^{-4}$ |
| 70 | cg24777950 | CTSG | 14 | 24115961 | - | -2.57 | (0.45) | $2.14 \times 10^{-8}$ | $4.91 \times 10^{-4}$ |
| 71 | cg14654385 | URP2 | 11 | 63729582 | + | -2.61 | (0.46) | $2.30 \times 10^{-8}$ | $5.27 \times 10^{-4}$ |
| 72 | cg10161121 | FASLG | 1 | 170894643 | + | 2.91 | (0.51) | $2.34 \times 10^{-8}$ | $5.36 \times 10^{-4}$ |
| 73 | cg21126943 | CEACAM6 | 19 | 46951235 | + | -2.85 | (0.50) | $2.37 \times 10^{-8}$ | $5.43 \times 10^{-4}$ |
| 74 | cg25028542 | ANLN | 7 | 36395645 | + | -4.12 | (0.73) | $2.45 \times 10^{-8}$ | $5.61 \times 10^{-4}$ |
| 75 | cg22534509 | GPR81 | 12 | 121781072 | - | 2.75 | (0.49) | $2.55 \times 10^{-8}$ | $5.85 \times 10^{-4}$ |
| 76 | cg27019278 | EMCN | 4 | 101657827 | - | -3.51 | (0.62) | $2.64 \times 10^{-8}$ | $6.04 \times 10^{-4}$ |
| 77 | cg02266731 | CPM | 12 | 67643600 | - | -3.40 | (0.60) | $2.67 \times 10^{-8}$ | $6.11 \times 10^{-4}$ |

| | | | | | | | | | |
|---|---|---|---|---|---|---|---|---|---|
| 78 | cg11098259 | AQP9 | 15 | 56217683 | + | -3.11 | ( 0.55 ) | $2.80 \times 10^{-8}$ | $6.41 \times 10^{-4}$ |
| 79 | cg27461196 | FXYD1 | 19 | 40321946 | + | -3.09 | ( 0.55 ) | $2.83 \times 10^{-8}$ | $6.50 \times 10^{-4}$ |
| 80 | cg17186163 | C10orf10 | 10 | 44794323 | - | -3.74 | ( 0.66 ) | $3.21 \times 10^{-8}$ | $7.36 \times 10^{-4}$ |
| 81 | cg24474182 | P2RY13 | 3 | 152529997 | - | -3.02 | ( 0.54 ) | $3.31 \times 10^{-8}$ | $7.59 \times 10^{-4}$ |
| 82 | cg22820108 | NCOR2 | 12 | 123569171 | - | -4.11 | ( 0.73 ) | $3.37 \times 10^{-8}$ | $7.73 \times 10^{-4}$ |
| 83 | cg02473123 | CD7 | 17 | 77868065 | - | 4.04 | ( 0.72 ) | $3.46 \times 10^{-8}$ | $7.93 \times 10^{-4}$ |
| 84 | cg21917349 | APBA2 | 15 | 27001152 | + | 3.37 | ( 0.60 ) | $3.72 \times 10^{-8}$ | $8.54 \times 10^{-4}$ |
| 85 | cg00071250 | FASLG | 1 | 170894886 | + | 2.55 | ( 0.46 ) | $3.83 \times 10^{-8}$ | $8.78 \times 10^{-4}$ |
| 86 | cg13030582 | MFAP4 | 17 | 19231301 | - | -3.45 | ( 0.62 ) | $4.06 \times 10^{-8}$ | $9.31 \times 10^{-4}$ |
| 87 | cg07658590 | SLC19A1 | 21 | 45788015 | - | -2.87 | ( 0.51 ) | $4.07 \times 10^{-8}$ | $9.32 \times 10^{-4}$ |
| 88 | cg21991396 | CIAS1 | 1 | 245648040 | + | -2.23 | ( 0.40 ) | $4.24 \times 10^{-8}$ | $9.71 \times 10^{-4}$ |
| 89 | cg13053608 | LGP1 | 17 | 37599199 | - | -2.67 | ( 0.48 ) | $4.24 \times 10^{-8}$ | $9.73 \times 10^{-4}$ |
| 90 | cg11827101 | LOC339789 | 2 | 8385873 | - | -3.67 | ( 0.66 ) | $4.31 \times 10^{-8}$ | $9.87 \times 10^{-4}$ |
| 91 | cg01965939 | SH3TC2 | 5 | 148422887 | - | -3.79 | ( 0.68 ) | $4.31 \times 10^{-8}$ | $9.89 \times 10^{-4}$ |
| 92 | cg13277939 | CTAGE5 | 14 | 38804962 | + | -3.18 | ( 0.57 ) | $4.72 \times 10^{-8}$ | $1.08 \times 10^{-3}$ |
| 93 | cg08700306 | LRP3 | 19 | 38378230 | + | -3.54 | ( 0.64 ) | $4.81 \times 10^{-8}$ | $1.10 \times 10^{-3}$ |
| 94 | cg08044694 | BRD4 | 19 | 15252927 | - | -2.32 | ( 0.42 ) | $4.94 \times 10^{-8}$ | $1.13 \times 10^{-3}$ |
| 95 | cg05859264 | MAPK13 | 6 | 36205670 | + | 3.46 | ( 0.63 ) | $5.11 \times 10^{-8}$ | $1.17 \times 10^{-3}$ |
| 96 | cg26105232 | IL2RA | 10 | 6145662 | - | -3.73 | ( 0.67 ) | $5.35 \times 10^{-8}$ | $1.23 \times 10^{-3}$ |
| 97 | cg02240622 | PLCB2 | 15 | 38388759 | - | -2.40 | ( 0.44 ) | $5.73 \times 10^{-8}$ | $1.31 \times 10^{-3}$ |
| 98 | cg19154438 | CKM | 19 | 50517570 | - | 3.87 | ( 0.70 ) | $5.84 \times 10^{-8}$ | $1.34 \times 10^{-3}$ |
| 99 | cg16509045 | TRPM6 | 9 | 76692783 | - | -2.78 | ( 0.51 ) | $6.13 \times 10^{-8}$ | $1.40 \times 10^{-3}$ |
| 100 | cg21019522 | SLC22A18 | 11 | 2877365 | + | -3.22 | ( 0.58 ) | $6.22 \times 10^{-8}$ | $1.43 \times 10^{-3}$ |
| 101 | cg11983245 | KRT15 | 17 | 36928680 | - | 5.49 | ( 1.00 ) | $6.45 \times 10^{-8}$ | $1.48 \times 10^{-3}$ |
| 102 | cg00899659 | ZNF22 | 10 | 44815977 | + | -2.98 | ( 0.54 ) | $6.67 \times 10^{-8}$ | $1.53 \times 10^{-3}$ |
| 103 | cg06317209 | AVIL | 12 | 56497145 | - | -3.51 | ( 0.64 ) | $7.05 \times 10^{-8}$ | $1.62 \times 10^{-3}$ |
| 104 | cg05000446 | MGC41945 | 9 | 35032395 | + | -2.98 | ( 0.54 ) | $7.06 \times 10^{-8}$ | $1.62 \times 10^{-3}$ |
| 105 | cg09624565 | NCF4 | 22 | 35586846 | + | -3.63 | ( 0.66 ) | $7.07 \times 10^{-8}$ | $1.62 \times 10^{-3}$ |
| 106 | cg21400896 | ABI3 | 17 | 44643568 | + | 3.61 | ( 0.66 ) | $7.16 \times 10^{-8}$ | $1.64 \times 10^{-3}$ |
| 107 | cg13471990 | ENTPD1 | 10 | 97505212 | + | -3.53 | ( 0.65 ) | $7.24 \times 10^{-8}$ | $1.66 \times 10^{-3}$ |
| 108 | cg13703437 | FYB | 5 | 39255455 | - | -2.60 | ( 0.48 ) | $7.30 \times 10^{-8}$ | $1.67 \times 10^{-3}$ |
| 109 | cg14324675 | LST1 | 6 | 31662827 | + | -2.98 | ( 0.54 ) | $7.48 \times 10^{-8}$ | $1.71 \times 10^{-3}$ |
| 110 | cg02600394 | TXK | 4 | 47830991 | - | 3.18 | ( 0.58 ) | $7.60 \times 10^{-8}$ | $1.74 \times 10^{-3}$ |
| 111 | cg10061138 | STAB1 | 3 | 52504125 | + | -3.55 | ( 0.65 ) | $7.63 \times 10^{-8}$ | $1.75 \times 10^{-3}$ |
| 112 | cg17839611 | GNGT2 | 17 | 44641801 | - | 3.31 | ( 0.61 ) | $7.68 \times 10^{-8}$ | $1.76 \times 10^{-3}$ |
| 113 | cg24427660 | PNPLA2 | 11 | 808892 | + | -2.37 | ( 0.44 ) | $8.63 \times 10^{-8}$ | $1.98 \times 10^{-3}$ |
| 114 | cg09076077 | FLJ33860 | 20 | 58063710 | + | -2.48 | ( 0.46 ) | $8.77 \times 10^{-8}$ | $2.01 \times 10^{-3}$ |
| 115 | cg06270401 | DYRK4 | 12 | 4569346 | + | -2.28 | ( 0.42 ) | $9.09 \times 10^{-8}$ | $2.08 \times 10^{-3}$ |
| 116 | cg17709873 | LTA | 6 | 31648435 | + | 2.91 | ( 0.54 ) | $9.16 \times 10^{-8}$ | $2.10 \times 10^{-3}$ |
| 117 | cg09914304 | PRF1 | 10 | 72032298 | - | 3.48 | ( 0.64 ) | $9.84 \times 10^{-8}$ | $2.26 \times 10^{-3}$ |

| | | | | | | | | | |
|---|---|---|---|---|---|---|---|---|---|
| 118 | cg27285056 | NAPSA | 19 | 55560691 | - | 3.54 | (0.66) | $1.03 \times 10^{-7}$ | $2.37 \times 10^{-3}$ |
| 119 | cg08539991 | ZBTB32 | 19 | 40895672 | + | 2.79 | (0.52) | $1.06 \times 10^{-7}$ | $2.43 \times 10^{-3}$ |
| 120 | cg00323915 | GIMAP4 | 7 | 149895920 | + | 3.11 | (0.58) | $1.06 \times 10^{-7}$ | $2.43 \times 10^{-3}$ |
| 121 | cg01402255 | GATAD2B | 1 | 152067323 | - | -2.66 | (0.49) | $1.09 \times 10^{-7}$ | $2.49 \times 10^{-3}$ |
| 122 | cg26701826 | MGC26963 | 4 | 109034053 | + | -2.53 | (0.47) | $1.11 \times 10^{-7}$ | $2.55 \times 10^{-3}$ |
| 123 | cg02374486 | PRF1 | 10 | 72032815 | - | 3.73 | (0.69) | $1.12 \times 10^{-7}$ | $2.57 \times 10^{-3}$ |
| 124 | cg24926276 | LRG1 | 19 | 4490943 | - | -2.71 | (0.50) | $1.16 \times 10^{-7}$ | $2.66 \times 10^{-3}$ |
| 125 | cg26540515 | ANGPT4 | 20 | 844349 | - | -3.99 | (0.74) | $1.19 \times 10^{-7}$ | $2.73 \times 10^{-3}$ |
| 126 | cg01813965 | C16orf50 | 16 | 56286605 | + | -2.86 | (0.53) | $1.24 \times 10^{-7}$ | $2.85 \times 10^{-3}$ |
| 127 | cg04759756 | SLA2 | 20 | 34707347 | - | 3.17 | (0.59) | $1.34 \times 10^{-7}$ | $3.08 \times 10^{-3}$ |
| 128 | cg00546897 | LOC284837 | 21 | 44056660 | - | -2.87 | (0.54) | $1.40 \times 10^{-7}$ | $3.22 \times 10^{-3}$ |
| 129 | cg20018806 | TCN1 | 11 | 59390450 | - | -4.12 | (0.77) | $1.40 \times 10^{-7}$ | $3.22 \times 10^{-3}$ |
| 130 | cg18350391 | IL32 | 16 | 3055553 | + | 3.15 | (0.59) | $1.43 \times 10^{-7}$ | $3.28 \times 10^{-3}$ |
| 131 | cg15739581 | GALNT3 | 2 | 166335029 | - | -3.17 | (0.59) | $1.48 \times 10^{-7}$ | $3.39 \times 10^{-3}$ |
| 132 | cg04451770 | ENTPD1 | 10 | 97505362 | + | -2.43 | (0.46) | $1.48 \times 10^{-7}$ | $3.40 \times 10^{-3}$ |
| 133 | cg09499849 | ACVR1 | 2 | 158403404 | - | -3.10 | (0.58) | $1.49 \times 10^{-7}$ | $3.42 \times 10^{-3}$ |
| 134 | cg16280667 | BLR1 | 11 | 118259803 | + | 4.89 | (0.92) | $1.53 \times 10^{-7}$ | $3.51 \times 10^{-3}$ |
| 135 | cg00415993 | F2RL2 | 5 | 75954944 | - | -2.33 | (0.44) | $1.60 \times 10^{-7}$ | $3.67 \times 10^{-3}$ |
| 136 | cg06172871 | HP | 16 | 70645745 | + | -2.63 | (0.50) | $1.62 \times 10^{-7}$ | $3.70 \times 10^{-3}$ |
| 137 | cg26928972 | CSTA | 3 | 123526489 | + | -2.83 | (0.53) | $1.69 \times 10^{-7}$ | $3.86 \times 10^{-3}$ |
| 138 | cg07730301 | ALDH3B1 | 11 | 67534528 | + | -3.05 | (0.58) | $1.71 \times 10^{-7}$ | $3.92 \times 10^{-3}$ |
| 139 | cg01623438 | CTSZ | 20 | 57016289 | - | -3.11 | (0.59) | $1.91 \times 10^{-7}$ | $4.38 \times 10^{-3}$ |
| 140 | cg07409200 | FLJ40919 | 13 | 42252674 | + | -2.90 | (0.55) | $1.92 \times 10^{-7}$ | $4.40 \times 10^{-3}$ |
| 141 | cg24091474 | TYROBP | 19 | 41091025 | - | -2.37 | (0.45) | $1.98 \times 10^{-7}$ | $4.53 \times 10^{-3}$ |
| 142 | cg17980508 | IFI44L | 1 | 78858301 | + | -2.89 | (0.55) | $2.02 \times 10^{-7}$ | $4.63 \times 10^{-3}$ |
| 143 | cg24453664 | CD59 | 11 | 33714989 | - | -2.34 | (0.44) | $2.03 \times 10^{-7}$ | $4.65 \times 10^{-3}$ |
| 144 | cg25226014 | CXCR6 | 3 | 45959946 | + | 2.96 | (0.56) | $2.19 \times 10^{-7}$ | $5.03 \times 10^{-3}$ |
| 145 | cg16592658 | EBI3 | 19 | 4180887 | + | 2.95 | (0.56) | $2.20 \times 10^{-7}$ | $5.04 \times 10^{-3}$ |
| 146 | cg26112639 | CIAS1 | 1 | 245646729 | + | -2.73 | (0.52) | $2.39 \times 10^{-7}$ | $5.47 \times 10^{-3}$ |
| 147 | cg19963522 | PIP3-E | 6 | 154719488 | - | -2.95 | (0.56) | $2.39 \times 10^{-7}$ | $5.49 \times 10^{-3}$ |
| 148 | cg18338021 | GZMM | 19 | 495349 | + | 3.98 | (0.76) | $2.41 \times 10^{-7}$ | $5.52 \times 10^{-3}$ |
| 149 | cg11340260 | GP1BA | 17 | 4776256 | + | -3.25 | (0.62) | $2.51 \times 10^{-7}$ | $5.76 \times 10^{-3}$ |
| 150 | cg19812619 | ITGB7 | 12 | 51886921 | - | 3.20 | (0.61) | $2.52 \times 10^{-7}$ | $5.77 \times 10^{-3}$ |
| 151 | cg01980222 | TREM2 | 6 | 41238895 | - | -3.25 | (0.62) | $2.53 \times 10^{-7}$ | $5.81 \times 10^{-3}$ |
| 152 | cg08130265 | C15orf5 | 15 | 75306225 | - | -3.06 | (0.58) | $2.58 \times 10^{-7}$ | $5.92 \times 10^{-3}$ |
| 153 | cg08837884 | LRG1 | 19 | 4491782 | - | -3.92 | (0.75) | $2.67 \times 10^{-7}$ | $6.11 \times 10^{-3}$ |
| 154 | cg21842274 | CRHBP | 5 | 76284393 | + | -3.85 | (0.74) | $2.73 \times 10^{-7}$ | $6.26 \times 10^{-3}$ |
| 155 | cg18638581 | HK2 | 2 | 74913110 | + | -2.54 | (0.49) | $2.74 \times 10^{-7}$ | $6.28 \times 10^{-3}$ |
| 156 | cg11721194 | SLAMF7 | 1 | 158975863 | + | 3.40 | (0.65) | $2.75 \times 10^{-7}$ | $6.30 \times 10^{-3}$ |
| 157 | cg14973995 | TETRAN | 4 | 2905490 | - | -2.08 | (0.40) | $2.76 \times 10^{-7}$ | $6.33 \times 10^{-3}$ |

| | | | | | | | | | |
|---|---|---|---|---|---|---|---|---|---|
| 158 | cg11432797 | SPN | 16 | 29581988 | + | -10.38 | ( 1.99 ) | $2.88 \times 10^{-7}$ | $6.60 \times 10^{-3}$ |
| 159 | cg15512851 | FGD2 | 6 | 37081496 | + | -2.68 | ( 0.52 ) | $2.88 \times 10^{-7}$ | $6.61 \times 10^{-3}$ |
| 160 | cg13504059 | CCR7 | 17 | 35975468 | - | 3.23 | ( 0.62 ) | $2.90 \times 10^{-7}$ | $6.64 \times 10^{-3}$ |
| 161 | cg17740645 | GRB7 | 17 | 35147939 | + | -2.32 | ( 0.44 ) | $2.90 \times 10^{-7}$ | $6.65 \times 10^{-3}$ |
| 162 | cg05252264 | FCAR | 19 | 60077399 | + | -2.92 | ( 0.56 ) | $2.92 \times 10^{-7}$ | $6.70 \times 10^{-3}$ |
| 163 | cg05961212 | ADPRH | 3 | 120780885 | + | -3.52 | ( 0.68 ) | $2.93 \times 10^{-7}$ | $6.72 \times 10^{-3}$ |
| 164 | cg13745346 | CBFA2T3 | 16 | 87572024 | - | -3.21 | ( 0.62 ) | $3.06 \times 10^{-7}$ | $7.01 \times 10^{-3}$ |
| 165 | cg13500819 | PACAP | 5 | 138753299 | - | 3.19 | ( 0.62 ) | $3.27 \times 10^{-7}$ | $7.50 \times 10^{-3}$ |
| 166 | cg18149207 | RORC | 1 | 150072258 | - | 5.04 | ( 0.97 ) | $3.33 \times 10^{-7}$ | $7.64 \times 10^{-3}$ |
| 167 | cg04536922 | FAM13A1 | 4 | 90197589 | - | -2.97 | ( 0.58 ) | $3.73 \times 10^{-7}$ | $8.55 \times 10^{-3}$ |
| 168 | cg07924575 | HPS4 | 22 | 25211231 | - | -3.34 | ( 0.65 ) | $3.73 \times 10^{-7}$ | $8.56 \times 10^{-3}$ |
| 169 | cg12943082 | CCL26 | 7 | 75257247 | - | -2.04 | ( 0.40 ) | $3.75 \times 10^{-7}$ | $8.59 \times 10^{-3}$ |
| 170 | cg12629325 | PCDHAC1 | 5 | 140286642 | + | -3.04 | ( 0.59 ) | $3.93 \times 10^{-7}$ | $9.01 \times 10^{-3}$ |
| 171 | cg17676574 | UROC1 | 3 | 127720063 | - | 4.19 | ( 0.82 ) | $4.09 \times 10^{-7}$ | $9.37 \times 10^{-3}$ |
| 172 | cg00431050 | ELOVL3 | 10 | 103975720 | + | -4.25 | ( 0.83 ) | $4.28 \times 10^{-7}$ | $9.80 \times 10^{-3}$ |
| 173 | cg12554857 | PGDS | 4 | 95483042 | - | -2.80 | ( 0.55 ) | $4.38 \times 10^{-7}$ | $1.01 \times 10^{-2}$ |
| 174 | cg25066857 | GNLY | 2 | 85774949 | + | 3.55 | ( 0.69 ) | $4.44 \times 10^{-7}$ | $1.02 \times 10^{-2}$ |
| 175 | cg00112517 | PPP1R1B | 17 | 35036537 | + | -4.63 | ( 0.91 ) | $4.67 \times 10^{-7}$ | $1.07 \times 10^{-2}$ |
| 176 | cg15528736 | FCGRT | 19 | 54707592 | + | -2.41 | ( 0.47 ) | $4.92 \times 10^{-7}$ | $1.13 \times 10^{-2}$ |
| 177 | cg17952262 | MFSD7 | 4 | 673240 | - | -3.42 | ( 0.67 ) | $5.36 \times 10^{-7}$ | $1.23 \times 10^{-2}$ |
| 178 | cg25587233 | PPP2R4 | 9 | 130911826 | + | -3.31 | ( 0.65 ) | $5.40 \times 10^{-7}$ | $1.24 \times 10^{-2}$ |
| 179 | cg20713492 | AQP10 | 1 | 152560106 | + | -3.36 | ( 0.66 ) | $5.47 \times 10^{-7}$ | $1.25 \times 10^{-2}$ |
| 180 | cg01367992 | LY9 | 1 | 159033159 | + | 2.67 | ( 0.53 ) | $5.52 \times 10^{-7}$ | $1.27 \times 10^{-2}$ |
| 181 | cg15784615 | LTBR | 12 | 6364028 | + | -2.75 | ( 0.54 ) | $5.58 \times 10^{-7}$ | $1.28 \times 10^{-2}$ |
| 182 | cg22088368 | MGC35206 | 22 | 35734834 | - | -4.00 | ( 0.79 ) | $5.65 \times 10^{-7}$ | $1.30 \times 10^{-2}$ |
| 183 | cg04653308 | PPP2R4 | 9 | 130911839 | + | -3.23 | ( 0.64 ) | $5.69 \times 10^{-7}$ | $1.30 \times 10^{-2}$ |
| 184 | cg19906550 | SLC22A18 | 11 | 2877375 | + | -3.02 | ( 0.60 ) | $5.75 \times 10^{-7}$ | $1.32 \times 10^{-2}$ |
| 185 | cg25165199 | RCBTB2 | 13 | 48005694 | - | -2.99 | ( 0.59 ) | $5.97 \times 10^{-7}$ | $1.37 \times 10^{-2}$ |
| 186 | cg05697976 | MLSTD1 | 12 | 29267750 | + | -3.00 | ( 0.59 ) | $6.26 \times 10^{-7}$ | $1.43 \times 10^{-2}$ |
| 187 | cg15518883 | SIT1 | 9 | 35640561 | - | 2.68 | ( 0.53 ) | $6.28 \times 10^{-7}$ | $1.44 \times 10^{-2}$ |
| 188 | cg05253159 | APS | 7 | 101715280 | + | -4.09 | ( 0.81 ) | $6.36 \times 10^{-7}$ | $1.46 \times 10^{-2}$ |
| 189 | cg21578541 | TLR9 | 3 | 52235130 | - | -3.18 | ( 0.63 ) | $6.40 \times 10^{-7}$ | $1.47 \times 10^{-2}$ |
| 190 | cg08445039 | FKBP9 | 7 | 32964184 | + | -3.65 | ( 0.72 ) | $6.42 \times 10^{-7}$ | $1.47 \times 10^{-2}$ |
| 191 | cg18390025 | ELOVL3 | 10 | 103976726 | + | -2.75 | ( 0.54 ) | $6.46 \times 10^{-7}$ | $1.48 \times 10^{-2}$ |
| 192 | cg06196379 | TREM1 | 6 | 41362863 | - | -3.31 | ( 0.66 ) | $6.91 \times 10^{-7}$ | $1.58 \times 10^{-2}$ |
| 193 | cg26581729 | NPDC1 | 9 | 139059613 | - | -2.38 | ( 0.47 ) | $6.95 \times 10^{-7}$ | $1.59 \times 10^{-2}$ |
| 194 | cg04183425 | ASF1A | 6 | 119262978 | + | 3.50 | ( 0.70 ) | $6.99 \times 10^{-7}$ | $1.60 \times 10^{-2}$ |
| 195 | cg05989054 | GAMT | 19 | 1353626 | - | -3.41 | ( 0.68 ) | $7.01 \times 10^{-7}$ | $1.61 \times 10^{-2}$ |
| 196 | cg10287137 | P2RY2 | 11 | 72606702 | + | -2.27 | ( 0.45 ) | $7.24 \times 10^{-7}$ | $1.66 \times 10^{-2}$ |
| 197 | cg18468842 | SLC13A3 | 20 | 44746516 | - | -3.46 | ( 0.69 ) | $7.24 \times 10^{-7}$ | $1.66 \times 10^{-2}$ |

| | | | | | | | | | |
|---|---|---|---|---|---|---|---|---|---|
| 198 | cg13553498 | CLEC2D | 12 | 9713253 | + | 2.94 | ( 0.59 ) | $7.62 \times 10^{-7}$ | $1.75 \times 10^{-2}$ |
| 199 | cg16872071 | RALGDS | 9 | 134986976 | - | -2.90 | ( 0.58 ) | $7.72 \times 10^{-7}$ | $1.77 \times 10^{-2}$ |
| 200 | cg26697117 | P2RX1 | 17 | 3767267 | - | -4.10 | ( 0.82 ) | $7.83 \times 10^{-7}$ | $1.80 \times 10^{-2}$ |
| 201 | cg19399532 | FLJ35530 | 1 | 176779118 | + | -2.45 | ( 0.49 ) | $7.97 \times 10^{-7}$ | $1.83 \times 10^{-2}$ |
| 202 | cg15227982 | C10orf26 | 10 | 104525844 | + | -2.62 | ( 0.52 ) | $8.08 \times 10^{-7}$ | $1.85 \times 10^{-2}$ |
| 203 | cg15361231 | GLRX2 | 1 | 191341814 | - | -2.54 | ( 0.51 ) | $8.14 \times 10^{-7}$ | $1.87 \times 10^{-2}$ |
| 204 | cg15743985 | CD22 | 19 | 40511649 | + | -2.74 | ( 0.55 ) | $8.24 \times 10^{-7}$ | $1.89 \times 10^{-2}$ |
| 205 | cg22933847 | MRGPRF | 11 | 68536712 | - | -3.30 | ( 0.66 ) | $8.51 \times 10^{-7}$ | $1.95 \times 10^{-2}$ |
| 206 | cg24898863 | S100A8 | 1 | 151630204 | - | -2.90 | ( 0.58 ) | $8.64 \times 10^{-7}$ | $1.98 \times 10^{-2}$ |
| 207 | cg00648883 | LOC196549 | 13 | 31422761 | + | -3.34 | ( 0.67 ) | $1.01 \times 10^{-6}$ | $2.32 \times 10^{-2}$ |
| 208 | cg05714729 | GALNT1 | 18 | 31488094 | + | -2.46 | ( 0.50 ) | $1.03 \times 10^{-6}$ | $2.37 \times 10^{-2}$ |
| 209 | cg15121304 | | 22 | 20710100 | + | 4.54 | ( 0.92 ) | $1.04 \times 10^{-6}$ | $2.37 \times 10^{-2}$ |
| 210 | cg02516189 | CARD9 | 9 | 138387913 | - | -3.88 | ( 0.78 ) | $1.04 \times 10^{-6}$ | $2.38 \times 10^{-2}$ |
| 211 | cg02332073 | TSGA13 | 7 | 130022959 | - | -2.85 | ( 0.58 ) | $1.08 \times 10^{-6}$ | $2.48 \times 10^{-2}$ |
| 212 | cg17142183 | IL1R2 | 2 | 101974624 | + | -2.51 | ( 0.51 ) | $1.08 \times 10^{-6}$ | $2.48 \times 10^{-2}$ |
| 213 | cg00666746 | SYDE1 | 19 | 15078781 | + | -2.86 | ( 0.58 ) | $1.09 \times 10^{-6}$ | $2.51 \times 10^{-2}$ |
| 214 | cg03852570 | C10orf33 | 10 | 100165096 | - | -3.34 | ( 0.68 ) | $1.11 \times 10^{-6}$ | $2.54 \times 10^{-2}$ |
| 215 | cg23696618 | SERPINB10 | 18 | 59734679 | + | -3.31 | ( 0.67 ) | $1.14 \times 10^{-6}$ | $2.61 \times 10^{-2}$ |
| 216 | cg07525077 | RNASE3 | 14 | 20429783 | + | -2.90 | ( 0.59 ) | $1.15 \times 10^{-6}$ | $2.64 \times 10^{-2}$ |
| 217 | cg06791867 | TSPAN18 | 11 | 44837520 | + | -3.90 | ( 0.79 ) | $1.17 \times 10^{-6}$ | $2.67 \times 10^{-2}$ |
| 218 | cg02415431 | IGLL1 | 22 | 22253255 | - | -4.18 | ( 0.85 ) | $1.19 \times 10^{-6}$ | $2.72 \times 10^{-2}$ |
| 219 | cg08529852 | EGFL7 | 9 | 138677741 | + | -2.80 | ( 0.57 ) | $1.20 \times 10^{-6}$ | $2.75 \times 10^{-2}$ |
| 220 | cg18538812 | GIF | 11 | 59370671 | - | 3.22 | ( 0.65 ) | $1.24 \times 10^{-6}$ | $2.84 \times 10^{-2}$ |
| 221 | cg01182697 | TMEM59 | 1 | 54293020 | - | -2.71 | ( 0.55 ) | $1.24 \times 10^{-6}$ | $2.85 \times 10^{-2}$ |
| 222 | cg11630392 | GPR171 | 3 | 152403654 | - | 3.61 | ( 0.73 ) | $1.25 \times 10^{-6}$ | $2.87 \times 10^{-2}$ |
| 223 | cg23571857 | BIRC4BP | 17 | 6599622 | + | -3.14 | ( 0.64 ) | $1.29 \times 10^{-6}$ | $2.97 \times 10^{-2}$ |
| 224 | cg18413900 | CYP27B1 | 12 | 56447256 | - | -3.57 | ( 0.73 ) | $1.31 \times 10^{-6}$ | $3.00 \times 10^{-2}$ |
| 225 | cg15146752 | EPHA2 | 1 | 16355354 | - | -2.28 | ( 0.47 ) | $1.32 \times 10^{-6}$ | $3.02 \times 10^{-2}$ |
| 226 | cg15376097 | EVA1 | 11 | 117640480 | - | -3.99 | ( 0.81 ) | $1.33 \times 10^{-6}$ | $3.04 \times 10^{-2}$ |
| 227 | cg20674521 | KCNJ4 | 22 | 37180467 | - | 4.48 | ( 0.92 ) | $1.33 \times 10^{-6}$ | $3.06 \times 10^{-2}$ |
| 228 | cg22424746 | VTCN1 | 1 | 117554836 | - | 3.87 | ( 0.79 ) | $1.37 \times 10^{-6}$ | $3.15 \times 10^{-2}$ |
| 229 | cg08598221 | SNTB1 | 8 | 121894110 | - | -2.98 | ( 0.61 ) | $1.40 \times 10^{-6}$ | $3.21 \times 10^{-2}$ |
| 230 | cg01441777 | CSNK1E | 22 | 37044362 | - | -2.31 | ( 0.47 ) | $1.41 \times 10^{-6}$ | $3.24 \times 10^{-2}$ |
| 231 | cg18392482 | AMDHD1 | 12 | 94862142 | + | -2.86 | ( 0.59 ) | $1.49 \times 10^{-6}$ | $3.42 \times 10^{-2}$ |
| 232 | cg08510456 | BSN | 3 | 49566012 | + | -2.38 | ( 0.49 ) | $1.54 \times 10^{-6}$ | $3.54 \times 10^{-2}$ |
| 233 | cg11024597 | ECRG4 | 2 | 106047843 | + | -2.30 | ( 0.47 ) | $1.59 \times 10^{-6}$ | $3.66 \times 10^{-2}$ |
| 234 | cg10409560 | FLJ23657 | 4 | 76700323 | + | 4.03 | ( 0.83 ) | $1.60 \times 10^{-6}$ | $3.67 \times 10^{-2}$ |
| 235 | cg03574571 | CD22 | 19 | 40512021 | + | -2.31 | ( 0.47 ) | $1.60 \times 10^{-6}$ | $3.68 \times 10^{-2}$ |
| 236 | cg10636246 | AIM2 | 1 | 157313597 | - | -3.21 | ( 0.66 ) | $1.61 \times 10^{-6}$ | $3.68 \times 10^{-2}$ |
| 237 | cg02490034 | MEST | 7 | 129913072 | + | -2.46 | ( 0.51 ) | $1.64 \times 10^{-6}$ | $3.77 \times 10^{-2}$ |

| | | | | | | | | |
|---|---|---|---|---|---|---|---|---|
| 238 | cg08223235 | BCL2 | 18 | 59054814 | - | -2.73 ( 0.56 ) | $1.65 \times 10^{-6}$ | $3.79 \times 10^{-2}$ |
| 239 | cg22854223 | CD82 | 11 | 44543081 | + | -3.77 ( 0.78 ) | $1.67 \times 10^{-6}$ | $3.83 \times 10^{-2}$ |
| 240 | cg13765961 | MS4A1 | 11 | 59979814 | + | 3.07 ( 0.63 ) | $1.68 \times 10^{-6}$ | $3.84 \times 10^{-2}$ |
| 241 | cg04655481 | GPR21 | 9 | 124836630 | + | -2.75 ( 0.57 ) | $1.74 \times 10^{-6}$ | $4.00 \times 10^{-2}$ |
| 242 | cg15625636 | GPR65 | 14 | 87542190 | + | 3.40 ( 0.70 ) | $1.76 \times 10^{-6}$ | $4.03 \times 10^{-2}$ |
| 243 | cg24870391 | CCL11 | 17 | 29636788 | + | 5.67 ( 1.17 ) | $1.79 \times 10^{-6}$ | $4.09 \times 10^{-2}$ |
| 244 | cg20720686 | POR | 7 | 75420817 | + | -2.26 ( 0.47 ) | $1.83 \times 10^{-6}$ | $4.20 \times 10^{-2}$ |
| 245 | cg10236239 | SULT1C2 | 2 | 108360946 | + | -2.88 ( 0.60 ) | $1.83 \times 10^{-6}$ | $4.20 \times 10^{-2}$ |
| 246 | cg16745604 | CASP10 | 2 | 201755704 | + | -2.45 ( 0.51 ) | $1.85 \times 10^{-6}$ | $4.25 \times 10^{-2}$ |
| 247 | cg21846903 | VTN | 17 | 23721408 | - | -2.57 ( 0.53 ) | $1.87 \times 10^{-6}$ | $4.30 \times 10^{-2}$ |
| 248 | cg17399166 | CD1D | 1 | 156416419 | + | 4.33 ( 0.90 ) | $1.88 \times 10^{-6}$ | $4.32 \times 10^{-2}$ |
| 249 | cg14088811 | SPI1 | 11 | 47356570 | - | -2.24 ( 0.47 ) | $1.91 \times 10^{-6}$ | $4.38 \times 10^{-2}$ |
| 250 | cg06394229 | LGALS4 | 19 | 43995615 | - | -2.84 ( 0.59 ) | $2.05 \times 10^{-6}$ | $4.70 \times 10^{-2}$ |
| 251 | cg05341878 | RIMS2 | 8 | 104900822 | + | 3.39 ( 0.70 ) | $2.06 \times 10^{-6}$ | $4.73 \times 10^{-2}$ |
| 252 | cg02635407 | SH3TC1 | 4 | 8251449 | + | -2.79 ( 0.58 ) | $2.07 \times 10^{-6}$ | $4.74 \times 10^{-2}$ |
| 253 | cg25149155 | BCL2L2 | 14 | 22845617 | + | -3.68 ( 0.77 ) | $2.08 \times 10^{-6}$ | $4.76 \times 10^{-2}$ |
| 254 | cg10126923 | NKG7 | 19 | 56567263 | - | -2.01 ( 0.42 ) | $2.10 \times 10^{-6}$ | $4.82 \times 10^{-2}$ |
| 255 | cg07300408 | RNASE11 | 14 | 20128200 | - | -3.61 ( 0.75 ) | $2.10 \times 10^{-6}$ | $4.82 \times 10^{-2}$ |
| 256 | cg16692277 | GUCY1B2 | 13 | 50538949 | - | -4.22 ( 0.88 ) | $2.17 \times 10^{-6}$ | $4.96 \times 10^{-2}$ |
| 257 | cg17141902 | NINJ1 | 9 | 94937037 | - | 4.60 ( 0.96 ) | $2.18 \times 10^{-6}$ | $4.99 \times 10^{-2}$ |

*: Chromosomal location is based on NCBI build 36.1.
